# The Results of Rigid Titanium Plate Reinforcement and Only Conventional Wire Methods in Sternal Fixation in Morbidly Obese Patients

**DOI:** 10.21470/1678-9741-2023-0145

**Published:** 2023-10-17

**Authors:** Cem Atik, Derya Atik

**Affiliations:** 1 Cardiovascular Surgery Clinic, Private New Life Hospital, Osmaniye, Turkey; 2 Department of Nursing, Faculty of Health Sciences, Osmaniye Korkut Ata University, Osmaniye, Turkey

**Keywords:** Sternotomy, Mediastinitis, Titanium, Case-Control Studies, Obesity, Morbid, Sternum, Morbidity, Drainage

## Abstract

**Introduction:**

In this study, it was aimed to compare the clinical results and complications of rigid titanium plate reinforcement and only conventional wire methods for sternum fixation in morbidly obese patients who underwent sternotomy for open-heart surgery.

**Methods:**

The study was planned as a retrospective case-control study. Morbidly obese patients who underwent open-heart surgery with median sternotomy between 2011 and 2021 were analyzed retrospectively.

**Results:**

There was no statistically significant difference between the two groups in terms of characteristics of the patients (*P*≥0.05). Sternal dehiscence, sternum revision, wound drainage, and mediastinitis were significantly less common in the titanium plate group (*P*≤0.05). There was no statistically significant difference between the groups in terms of 30-day mortality (*P*≥0.05).

**Conclusion:**

Rigid titanium plate reinforcement application produced more positive clinical results than only conventional wire application. In addition, it was determined that although the rigid titanium plate application prolonged the operation time, it did not make a significant difference in terms of mortality and morbidity compared to the conventional wire applied group.

## INTRODUCTION

Median sternotomy is the most commonly used method to reach the mediastinum in cardiac surgery. When this method is used, serious complications such as sternal instability, dehiscence, and mediastinitis can be seen^[1]^. One of the most important reasons increasing the incidence of postoperative complications is obesity^[2]^. In addition, obesity is among the risk factors for sternal dehiscence and wound complications^[3,4]^.

Obesity causes conditions such as dyslipidemia, diabetes, hypertension, and inflammation, which are risk factors for coronary artery disease (CAD)^[5]^. The strong relationship between obesity and CAD increases the number of obese patients requiring surgical intervention day by day^[6]^.

A good sternal fixation should be done to reduce the complications of sternotomy, especially in morbidly obese patients. While choosing between sternal closure techniques, the correct evaluation according to the characteristics of the patient and surgical experience plays a very important role^[7]^. Morbidly obese patients who underwent median sternotomy during cardiac surgery may have a higher than normal risk of complications^[8]^. Rigid titanium plate fixation is one of the new sternal closure methods^[9]^. The rigid titanium plate fixation system consists of a transverse or longitudinal titanium plate fixed with self-tapping uni-lock screws^[10]^.

When the literature is examined, it is seen that sternal healing and clinical outcomes are better in patients who underwent rigid titanium plate fixation. However, there are limited studies evaluating the outcome of rigid titanium plate reinforcement application in terms of morbidly obese patients^[4,11,12,13]^.

In this study, it was aimed to present the clinical results of rigid titanium plate reinforcement and only conventional wire methods for sternum fixation in morbidly obese patients who underwent open-heart surgery with median sternotomy, thus contributing to the literature and the determination of the methods to be used for fixation in sternotomy.

## METHODS

### Study Design

This is a retrospective case-control study carried out in a private hospital in the south of Turkey.

Morbidly obese patients who underwent open-heart surgery with median sternotomy between 2011 and 2021 were analyzed retrospectively. The morbidly obese patients who underwent surgery before 2018 and were fixed with only conventional wire were in group 1, and morbidly obese patients who underwent fixation using conventional wire application in addition to rigid titanium plate reinforcement after 2018 were in group 2.

The results of the patients who met the inclusion criteria were compared. Inclusion criteria were:

1. Body mass index (BMI) ≥ 40 kg/m^2^
2. Patients on ventilator for ≤ 24 hours
3. Patients who do not use narcotic or similar before surgery
4. Patients without postoperative unconsciousness (stroke, etc.)
5. Reoperations

### Study Population and Data Collection

Sampling was not done to determine the patients to be included in the study. Data from all patients who met the inclusion criteria were used. The patients’ results of open-heart surgeries performed by CA, a cardiovascular surgeon working in the relevant hospital since 2011, and his team were evaluated. Data were obtained from the patient registry system.

Group 1 consisted of 247 morbidly obese patients who were applied only the conventional wire method and met the inclusion criteria, among 2,324 patients who underwent open-heart surgery between 2011 and 2018.

Group 2 consisted of 121 morbidly obese patients who were applied titanium plate reinforcement in addition to the conventional wire method and met the inclusion criteria, among 1,380 patients who underwent open-heart surgery between 2018 and 2022.

### Surgical Procedure

**Fig. 1:**
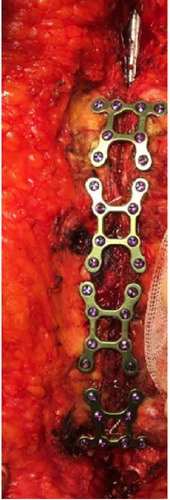
Rigid titanium plate reinforcement.

Only the classical conventional wire method was applied for sternum fixation in morbidly obese patients before 2018. In addition to conventional wire application, rigid titanium plate reinforcement was applied to morbidly obese patients after 2018. After 2018, standard interrupted eight-shaped closure with stainless steel wires was applied to all patients. The rigid titanium plate reinforcement consisted of titanium plates fixed with screws. Medplates 2.4 mm titanium sternum plate and screw set were used in the operations. At least two, at most four plates were used for the patients (Figure 1).

Patient-controlled analgesia was given to all patients immediately after extubation until they were removed from the surgical intensive care unit. None of the patients received continuous basal morphine infusion.

### Ethical Considerations

Permission was obtained from the hospital. The study was approved by the Scientific Research and Publication Ethics Committee of Osmaniye Korkut Ata University (11.11.2022/2022-9-6). Patient consent was not obtained because retrospective data were used in the study.

### Statistical Analysis

The data were analyzed using the IBM Corp. Released 2012, IBM SPSS Statistics for Windows, Version 21.0, Armonk, NY: IBM Corp. software at a significance level of 0.05. Mean ± standard deviation was used for continuous data. Frequency (n) and percentage (%) were used for categorical variables. Independent samples *t*-test and Chi-square test were used to compare the groups.

## RESULTS

The characteristics of the patients included in the study are presented in Table 1.

**Table 1:** Characteristics of the patients.

	Conventional wire + rigid titanium plate reinforcement applied group (n=121)	Conventional wire group (n=247)	P-value
Age, years (mean)	62 ± 5.16	59 ± 6.72	0.36
Gender, n (%)			
Female	91 (75.20)	186 (75.30)	
Male	30 (24.80)	61 (24.70)	1.18
Body mass index, ≥ 40 kg/m^2^ (mean)	41.1 ± 2.1	40.9 ± 1.9	0.82
Diabetes mellitus, n (%)	112 (92.56)	218 (88.25)	0.42
Peripheral vascular disease, n (%)	5 (4.13)	8 (3.24)	0.38
Hypertension, n (%)	98 (80.99)	193 (78.14)	0.21
COPD, n (%)	34 (28.09)	59 (23.88)	0.88
Smoker, n (%)	88 (72.72)	179 (72.46)	1.28
Creatinine level, mg/dL (mean)	1.16 ± 0.3	1.11 ± 0.2	0.15
Ejection fraction, % (mean)	51 ± 10.1	50 ± 8.8	0.99

COPD=chronic obstructive pulmonary disease

Data were presented as mean ± standard deviation or percent

The patients included in the study — in the rigid titanium plate reinforcement group and in the conventional wire group only — were evaluated according to their clinical and personal characteristics. There was no statistically significant difference between the two groups in terms of the patients’ characteristics (*P*≥0.05) (Table 1).

Intraoperative and postoperative clinical features of the patients are presented in Table 2.

**Table 2:** Intraoperative and postoperative clinical features.

	Conventional wire + rigid titanium plate reinforcement applied group (n=121)	Conventional wire group (n=247)	P-value
Isolated CABG, n (%)	88 (72.72)	182 (73.68)	0.34
Isolated AVR, n (%)	7 (5.79)	13 (5.27)	2.12
Isolated MVR, n (%)	6 (4.96)	14 (5.66)	1.28
AVR + MVR, n (%)	3 (2.48)	7 (2.84)	1.53
CABG + AVR, n (%)	10 (8.26)	21 (8.50)	1.21
CABG + MVR, n (%)	7 (5.79)	10 (4.05)	0.08
Cardiopulmonary bypass time, min (mean)	138 ± 51.21	133 ± 41.55	0.08
Myocardial ischemic time, min (mean)	96 ± 42.23	91 ± 32.41	0.07
Time spent in the operating room, min (mean)	345 ± 62.65	302 ± 50.31	0.06
Plate application time, min (mean)	33 ± 23.67	–	
Sternum revision, n (%)	1 (0.83)	22 (8.90)	**0.04**
Wound drainage, n (%)	2 (1.65)	18 (7.29)	**0.05**
Sternal dehiscence, n (%)	3 (2.48)	28 (11.33)	**0.04**
Mediastinitis, n (%)	0 (0)	4 (1.62)	**0.05**
30-day mortality, n (%)	4 (3.30)	7 (2.83)	0.09

AVR=aortic valve replacement; CABG=coronary artery bypass grafting; MVR=mitral valve replacement

Data were presented as mean ± standard deviation or percent

The groups were evaluated in terms of intraoperative and postoperative clinical features. There was no statistically significant difference between the groups in terms of intraoperative characteristics (*P*≥0.05). Statistically significant differences were found between the groups in terms of postoperative sternal dehiscence, sternum revision, wound drainage, and mediastinitis development. These complications were significantly less common in the rigid titanium plate reinforcement group (*P*≤0.05). There was no statistically significant difference between the groups in terms of 30-day mortality (*P*≥0.05) (Table 2).

When the results in the conventional wire + rigid titanium plate reinforcement applied group (group 2) and the only conventional wire applied group (group 1) were examined in terms of complications with a statistically significant difference, sternum revision was seen in 8.90% of patients in group 1 and 0.83% of patients in group 2, wound drainage was seen in 7.29% of patients in group 1 and 1.65% of patients in group 2, sternal dehiscence was seen in 11.33% of patients in group 1 and 2.48% of patients in group 2, and mediastinitis was seen in 1.62% of patients in group 1, but not in group 2. Although there was no statistical difference, the 30-day mortality rate was 2.83% in group 1 and 3.30% in group 2 (Table 2).

## DISCUSSION

In this study, the results of rigid titanium plate reinforcement and conventional wire methods applied in open-heart surgeries performed in a single clinic and by a single surgeon for morbidly obese patients were evaluated.

Sternal dehiscence, sternum revision, wound drainage, and mediastinitis development were statistically significantly less in the rigid titanium plate supplementation group in this study.

Synder et al. (2009)^[4]^ shared the rigid plate and wire application results of 129 high-risk patients with obesity, manual laborer, osteoporotic sternum, or intraoperative transverse sternal fracture. They stated that no early sternal wound complications were observed in the rigid plated group, late sternal wound complications were equal in both groups, and that primary rigid plating was beneficial compared to wire closure in the early postoperative period. The results of this study, in which risky groups including obese patients were evaluated, support our study results.

In the literature, there are not many studies evaluating the rigid titanium plate reinforcement and only conventional wire applications by taking morbidly obese patients as criteria. In studies conducted with different evaluation criteria, generally positive results of rigid titanium plate application were mentioned. In a randomized controlled study evaluating the six-month results of 236 patients by Allen et al. (2017)^[12]^, it was seen that the rigid plate group had better sternal healing and a lower complication rate than the wire applied group. In a meta-analysis including three randomized controlled studies and five observational studies, it was stated that there was no significant difference in terms of sternal complications between the rigid fixation and wire applied groups, and if there was no statistical significance, the complications were less in the rigid plate group^[13]^. The results of these studies are not for obese patients, but they support our study results.

Vos et al. (2017)^[14]^ retrospectively analyzed the results of 42 patients who underwent refixation after sternal dehiscence and underwent secondary wound closure due to the development of mediastinitis after sternotomy. They stated that rigid titanium plate fixation is superior to conventional refixation methods in stabilizing the sternal bone, especially in secondary wound closure of patients who developed mediastinitis after sternotomy. Although only the groups that developed complications were evaluated in this study, the results of the procedure seem to be in line with our results.

In the study of Tanyeli (2019)^[15]^, patients who developed sternum dehiscence after open-heart surgery were examined, and it was stated that rigid titanium plate application in patients with a mean BMI of 31.52 kg/m^2^ was particularly effective in comminuted fractures without stable intercostal spaces. Allen et al. (2018)^[16]^ examined 116 patients who underwent sternal rigid plate fixation and 120 patients who underwent wire cerclage, and it was stated that reduction in sternal pain and improvement in upper extremity function were better in patients with rigid plate. Liao et al. (2019)^[8]^ stated in their study that titanium plate application in primary closure is a suitable option for morbidly obese patients with a high risk of developing sternal dehiscence. Huh et al. (2008)^[17]^ stated in their study that titanium plate application is a more effective method than wire closure in patients with fractures, chronic and acute instability, or poor bone quality. And Kim et al. (2013)^[9]^ evaluated 17 patients with sternal dehiscence; they stated that titanium plate fixation with appropriate debridement and flap interposition is very effective in the treatment of patients who develop sternal dehiscence following major cardiac surgery. In these studies, the positive results of rigid plate application were mentioned.

### Limitations

In this study, the results of single-center one-team applications were presented.

## CONCLUSION

Sternal dehiscence, sternum revision, wound drainage, and mediastinitis were less common in patients who underwent sternum fixation with rigid titanium plate reinforcement in addition to conventional wire application after open-heart surgery with sternotomy. Although rigid titanium plate reinforcement slightly extended the operation time, it did not create a significant difference in mortality and morbidity compared to the conventional wire group. It will be more effective to strengthen our results if they are supported by the literature with randomized controlled studies to be conducted in obese and other patient groups.

## Abbreviations, Acronyms & Symbols

AVR: Aortic valve replacement
BMI: Body mass index
CABG: Coronary artery bypass grafting
CAD: Coronary artery disease
COPD: Chronic obstructive pulmonary disease
MVR: Mitral valve replacement
